# Modulation of matrix metalloproteases by ciliary neurotrophic factor in human placental development

**DOI:** 10.1007/s00441-022-03658-1

**Published:** 2022-07-07

**Authors:** Giovanni Tossetta, Sonia Fantone, Elena Marinelli Busilacchi, Nicoletta Di Simone, Stefano R. Giannubilo, Giovanni Scambia, Antonio Giordano, Daniela Marzioni

**Affiliations:** 1 Department of Experimental and Clinical Medicine, Università Politecnica Delle Marche, 60126 Ancona, Italy; 2 Clinica Di Ostetricia E Ginecologia, Azienda Ospedaliero Universitaria Ospedali Riuniti Di Ancona, 60123 Ancona, Italy; 3 Department of Clinical and Molecular Sciences, Università Politecnica Delle Marche, Ancona, Italy; 4 Hematology Unit, AUO Ospedali Riuniti Di Ancona, 60123 Ancona, Italy; 5 Department of Biomedical Science, Humanitas University, Via Rita Levi Montalcini 4, 20090 Pieve Emanuele, Milan, Italy; 6 Humanitas Clinical and Research Center-IRCCS, Via Manzoni56, 20089 Rozzano, Italy; 7 Department of Clinical Sciences, Università Politecnica Delle Marche, Salesi Hospital, 60123 Ancona, Italy; 8 U.O.C. Di Ostetricia E Patologia Ostetrica, Dipartimento Di Scienze Della Salute Della Donna, Fondazione Policlinico Universitario A. Gemelli IRCCS, del Bambino E Di Sanità Pubblica, 00168 Rome, Italy; 9 Istituto Di Clinica Ostetrica E Ginecologica, Università Cattolica del Sacro Cuore, 00168 Rome, Italy

**Keywords:** Placenta, MMP, CNTF, pSTAT3, pERK

## Abstract

Ciliary neurotrophic factor (CNTF) is a pleiotropic cytokine that signals through a receptor complex containing a specific subunit, CNTF receptor α (CNTFRα). The two molecules are constitutively expressed in key structures for human placental growth and differentiation. The possible role of CNTF in enhancing cell proliferation and/or invasion during placental development and remodelling was investigated using HTR-8/SVneo and BeWo cells, taken respectively as cytotrophoblast and syncytiotrophoblast models. In both cell lines, treatment with human recombinant (hr) CNTF activated JAK2/STAT3 signalling and inhibited the ERK pathway. Interestingly, in HTR-8/SVneo cells, 50 ng hrCNTF induced significant downregulation of matrix metalloprotease (MMP)-1 and significant upregulation of MMP-9. Moreover, pharmacological inhibition of JAK2/STAT3 signalling by AG490 and curcumin resulted in MMP-9 downregulation; it activated the ERK signalling pathway and upregulated MMP-1 expression. Collectively, these data suggest a role for CNTF signalling in extravillous cytotrophoblast invasion through the modulation of specific MMPs.

## Introduction

The development and differentiation of the human placental villous tree are regulated by numerous growth factors, their receptors and other types of molecules, such as matrix metalloproteases (MMPs) (Chen and Khalil 2017), whose balanced action is critical for normal placental development and successful pregnancy. MMPs play an important role in the degradation and remodelling of the decidual extracellular matrix and are critical for trophoblast adhesion, invasion and proliferation (Espino et al. 2017). MMPs are a large family of zinc-dependent endopeptidases that include gelatinases (e.g. MMP-2, MMP-9), collagenases (e.g. MMP-1, MMP-8 and MMP-13), matrilysins (e.g. MMP-7, MMP-26), stromelysins (e.g. MMP-3, MMP-10) and type I and II transmembrane metalloproteinases (Nagase et al. 2006). In particular, altered MMP-1, -2, -3 and -9 regulation during placentation may lead to placental disease such as pregnancy loss (Balci and Ozdemir 2019), preterm delivery (Sundrani et al. 2012), preeclampsia (PE) (Sahay et al. 2018) and intra-uterine growth restriction (IUGR) (Swierczewski et al. 2012). A role for ciliary neurotrophic factor (CNTF) in MMP modulation has been demonstrated in murine neuroblastoma cell models, where CNTF downregulated MMP-2, thus potentially to affecting invasive behaviour (Sartor et al. 2002). CNTF is a 22-kDa cytokine encoded by the *CNTF* gene located on human chromosome 11 (Lam et al. 1991). It belongs to the interleukin (IL)-6 cytokine family along with cardiotrophin-1, IL-6, IL-11, IL-27, leukaemia inhibitory factor (LIF) and oncostatin-M (Heinrich et al. 2003). All the members of this family share glycoprotein 130 (gp130) as a signal-transducing receptor protein (Rose-John 2015). At the cellular level, the effects of CNTF are mediated via a receptor complex consisting of gp130, LIF receptor ß (LIFRß) and CNTF receptor α (CNTFRα) (Wagener et al. 2014). CNTFRα is widely expressed in neuronal cells (Ding et al. 2013), hepatocytes (Wang and Fuller 1995), adipocytes (Perugini et al. 2019), muscle (Steinberg et al. 2009), retinal (Ghasemi et al. 2018) and prostatic cells (Fantone et al. 2020b) and pancreatic β-cells (Rezende et al. 2009), where it plays key and complex roles in cell proliferation and differentiation and helps cells survive pathological stimuli (Ding et al. 2013).

The role of the IL-6 cytokine family in pregnancy has been extensively investigated (Ge et al. 2018; Horita et al. 2007; Markert et al. 2011). Interestingly, IL-6 and/or IL-6R dysregulation has been associated with impaired embryo implantation and abortion (Markert et al. 2011). In addition, IL-6 plays a pivotal role in regulating the proliferation and invasiveness of cytotrophoblast cells by increasing MMP-9 and MMP-2 activity (Meisser et al. 1999).

The IL-6 family members, particularly CNTF, can trigger different intracellular signalling events that depend on cell type, including activation of extracellular signal-regulated kinases (ERK) (Trimarchi et al. 2009), of the phosphatidylinositol 3 kinase (PI3K)-AKT pathway (Hiatt et al. 2014) and of the Janus kinase (JAK)2/signal transducer and activator of transcription (STAT)-3 signalling pathway (Rezende et al. 2009). Notably, CNTF can simultaneously activate multiple signalling pathways in the same cell type (Steinberg et al. 2009; Trimarchi et al. 2009).

Very few studies have explored a role for CNTF in pregnancy. A study of the circulating levels of CNTF in non-pregnant and pregnant women and in women with pregnancies complicated by PE has found that CNTF levels were lower in pregnant compared with non-pregnant women, that maternal CNTF blood levels tended to decline with the progression of normal pregnancy and that they were lower in pregnancies complicated by PE than in normal pregnancies (Akahori et al. 2010). In contrast, Bienertova-Vasku and colleagues showed no difference in maternal CNTF blood levels between normal and PE pregnancies at term, but described higher CNTF levels in umbilical cord foetal blood of PE compared with normal pregnancies (Bienertova-Vasku et al. 2013). Unfortunately, in the latter study, the samples were not matched for gestational age, thus preventing all comparison. We hypothesize that the lower CNTF levels found in maternal plasma during gestation and the altered CNTF levels described in pregnancies complicated by PE can be related to placental development during gestation. This study was devised to assess the presence of CNTF in maternal blood and understand its action on placental development. First we identified the placental structures expressing CNTF and CNTFRα in first- and third-trimester human placentas and then investigated CNTF involvement in placental development by testing its possible modulation of MMP-1, MMP-2 and MMP-9 (Chen and Khalil 2017).

## Materials and methods

### Tissue collection

We examined 24 placentas, 12 from pregnant women undergoing voluntary pregnancy termination at 9–12 weeks of gestation (Obstetrics and Gynaecology of San Severino Hospital, Macerata, Italy) and 12 from third-trimester pregnancies that were terminated by caesarean section (Department of Woman and Child Health, A. Gemelli Hospital, Università Cattolica del Sacro Cuore Rome, Italy and Obstetrics and Gynaecology, Department of Clinical Sciences, Polytechnic University of Marche, Ancona, Italy). In parallel, we also analysed first-trimester placentas from clinically normal pregnancies interrupted by aspiration curettage for psychosocial or medical reasons that were unlikely to affect placental structure or function. All women gave their written informed consent to the collection of placental specimens. The collection procedures were in accordance with the Helsinki Declaration of 1975, as revised in 2013. The permission of the Human Investigation Committee of the Marche Region (Italy) was obtained (protocol no. 2019.172; study ID 980; CERM no. 172).

Immediately after delivery and gross examination, three zones were identified in each placenta: a central zone near the umbilical cord insertion, a peripheral zone, i.e. the most distal from the umbilical cord, and an intermediate zone. Two tissue samples were collected from each zone. The samples for immunohistochemistry were fixed in 4% buffered formalin at 4 °C for 12 h and routinely processed for paraffin embedding at 56° C, whereas the samples for biochemical and molecular analysis were frozen in liquid nitrogen as described previously (Fantone et al. 2020a; Tossetta et al. 2019).

### Immunohistochemical analysis of first- and third-trimester placentas

Immunohistochemical staining was performed as described previously (Altobelli et al. 2017; Avellini et al. 2017). Briefly, after dewaxing, paraffin sections were rinsed in phosphate buffered saline (PBS) and incubated with 3% hydrogen peroxide for 40 min to block endogenous peroxidase. They were then subjected to high-temperature pretreatment in 10 mM citrate buffer, pH 6.0, for 5 min (CNTF) or in 100 ng/ml proteinase K (Sigma‐Aldrich, St. Louis, MO, USA) for 5 min at 37 °C (CNTFRα). Sections were subsequently rinsed with PBS and incubated with normal horse serum (Vector Laboratories, Burlingame, CA, USA) diluted 1:75 in PBS for 1 h at room temperature (RT). This was followed by incubation with the primary antibodies (Table 1) diluted in PBS and then overnight incubation at 4 °C. After a thorough rinse in PBS, sections were incubated in a 1:200 biotinylated secondary antibody (Vector Laboratories) solution for 30 min at RT. Histochemical reactions were performed using Vectastain ABC Kit (Vector Laboratories) for 1 h at RT with 3′,3′‐ diaminobenzidine hydrochloride (Sigma‐Aldrich) as the chromogen. Sections were counterstained with Mayer’s haematoxylin, dehydrated and mounted in Eukitt (Kindler GmbH and Co., Freiburg, Germany). Negative controls were performed by replacing the primary or the secondary antibody with an isotype antibody: monoclonal rabbit IgG (ab172730; Abcam, Cambridge, UK) for CNTF and monoclonal mouse IgG2a (ab18415; Abcam) for CNTFRα.

**Table 1:** List of antibodies

Antibody	IHC	Wb	IF	Company
pAb Rabbit anti-human CNTF (#PA1-18,358)	//	1:400	//	Thermo Fisher Scientific, MA, USA
pAb Rabbit anti-human CNTF (#ab190985)	1:100	//	1:150	Abcam, Cambridge, UK
pAb Rabbit anti-human CNTFRα (#PA5-45,053)	//	1:400	//	Thermo Fisher Scientific, MA, USA
mAb Mouse anti-human CNTFRα (#ab89333)	1:150	//	1:100	Abcam, Cambridge, UK
mAb Rabbit anti-human pAKT (#4060)	//	1:1000	//	Cell Signaling Technology, MA, USA
pAb Rabbit anti-human AKT (#9272)	//	1:1000	//	Cell Signaling Technology, MA, USA
mAb Rabbit anti-human pERK1/2 (#4377)	//	1:800	//	Cell Signaling Technology, MA, USA
mAb Rabbit anti-human ERK1/2 (#4695)	//	1:1000	//	Cell Signaling Technology, MA, USA
mAb Mouse anti-human pSTAT3 (#4113)	//	1:800	//	Cell Signaling Technology, MA, USA
mAb Rabbit anti-human STAT3 (#4904)	//	1:1000	//	Cell Signaling Technology, MA, USA
mAb Mouse anti-human PCNA (#sc-56)	//	1:600	//	Santa Cruz Biotechnology, TX, USA
pAb Rabbit anti-human MMP1 (#PA5-16,498)	//	1:300	//	Thermo Fisher Scientific, MA, USA
mAb Mouse anti-human MMP2 (#436,000)	//	1:300	//	Thermo Fisher Scientific, MA, USA
pAb Rabbit anti-human MMP9 (#10,375–2-AP)	//	1:500	//	Proteintech, Rosemont, USA
mAb Mouse anti-human ACTIN (#sc-8432)	//	1:1000	//	Santa Cruz Biotechnology, TX, USA

### Culture of HTR-8/SVneo and BeWo cell lines

To test the involvement of CNTF in proliferation and invasion processes, we used the cytotrophoblast cell line HTR-8/SVneo (kindly provided by C. H. Graham, Queen’s University, Kingston, ON, Canada) and the choriocarcinoma derived BeWo human placental cell line (kindly provided by S. Alberti, Laboratory of Cancer Pathology, CeSI-MeT, University ‘G. d’Annunzio’, Chieti, Italy). BeWo cells express an epithelial proteome characteristic of villous trophoblasts, whereas HTR-8/SVneo cells exhibit a phenotype characteristic of extravillous trophoblasts (Szklanna et al. 2017). Notably, BeWo cells also show a spontaneous ability to form syncytial units mimicking the syncytiotrophoblast, whereas a marked self-renewal ability makes HTR-8/SVneo cells a good model of cytotrophoblast cells (Weber et al. 2013). Although freshly isolated cytotrophoblast can differentiate into multinucleated syncytiotrophoblast, their inability to proliferate in vitro hampers experiments and prevents their use (Chen et al. 2013; King et al. 2000; Kohli et al. 2016; Mannelli et al. 2015).

HTR-8/SVneo cells (Graham et al. 1993) were cultured in RPMI 1640 medium (Life Technologies, Monza, Italy), whereas BeWo cells were cultured in DMEM/F12 medium. Both media were supplemented with 10% foetal bovine serum (FBS) and 100 U/ml penicillin and streptomycin (all from Gibco, Thermo Fisher Scientific, Waltham, MA, USA). All cultures were incubated at 37 °C with 95% humidity and 5% CO_2_. The culture media were changed twice a week.

### Immunofluorescence of HTR-8/SVneo and BeWo cell lines

HTR-8/SVneo and BeWo cells were washed in Dulbecco’s PBS (Life Technologies, Monza, Italy), fixed in 4% paraformaldehyde in PBS for 10 min at 4 °C and permeabilized in 0.1 M PBS added with 0.1% Triton X-100 (Sigma, Milano, Italy) for 5 min. After washing in PBS at RT, cells were blocked with 10% normal donkey serum (Jackson ImmunoResearch, Cambridgeshire, UK) in 0.1 M PBS and incubated overnight at 4 °C with anti-human CNTF and CNTFRα antibodies (Table 1). Cells were then washed 3 times in PBS and incubated with Alexa Fluor 488-conjugated donkey anti-rabbit (for CNTF) and Alexa Fluor 555-conjugated donkey anti-mouse (for CNTFRα) IgG secondary antibody (both from Thermo Fisher Scientific) for 30 min at RT. The TOTO3 probe was used for nuclear staining. Finally, the slides were cover-slipped with propyl gallate and evaluated with an Eclipse E600 fluorescence microscope (Nikon, Düsseldorf, Germany).

### Treatment of HTR-8/SVneo and BeWo cell lines with rhCNTF

Both placental cell lines express CNTFRα. After seeding at confluence (1.5 10^4^/cm^2^), they were incubated with 20, 50 or 100 ng/ml recombinant human (rh) CNTF (cat. #257-NT-010, R&D Systems, Minneapolis, MN, USA) for 15 min, to investigate the possible signalling pathways triggered by CNTF. We focused on 3 possible pathways where the IL-6 cytokine family is commonly involved, the JAK2/STAT3, PI3K/AKT and MAPK/ERK pathways.

The two cell lines were also treated with two pSTAT3 inhibitors, AG490 and curcumin (Merck, Darmstadt, Germany), to assess the specificity of CNTF action. To do so, HTR-8/SVneo cells were incubated with 100 µM AG490 or 15 µM curcumin with/without 50 ng/ml rhCNTF for 24 h in suitable media. The doses were chosen after evaluation of pSTAT3 inhibition in a dose–response curve.

Viable counts were routinely performed using the trypan blue dye exclusion test. All experiments were conducted in triplicate and were repeated at least 3 times.

### Protein extracts and Western blotting of human placental samples and cell lines

After thawing, the placental samples were washed with 0.1 M PBS, pH 7.4. Each sample (300 mg) was homogenized in an Ultra Turrax T8 homogenizer (IKA-WERKE, Lille, France) using a lysis buffer consisting of 0.1 M PBS, 0.1% (w/v) SDS, 1% (w/w) NONIDET‐P40, 1 mM (w/v) Na orthovanadate, 1 mM (w/w) phenyl methane sulfonyl fluoride (PMSF), 12 mM (w/v) Na deoxycholate and 1.7 μg/ml aprotinin, pH 7.5. The placenta samples and the treated and untreated HTR-8/SVneo and BeWo cells were lysed in lysis buffer, centrifuged at 10,000 g for 20 min at 4 °C, aliquoted and stored at − 80 °C until use. Protein concentrations were determined by a Bradford protein assay (Bio‐Rad Laboratories, Milano, Italy). All protein samples were fractionated on 10% SDS–polyacrylamide gels (SDS-PAGE) as described previously (Laemmli 1970), electrophoretically transferred (Trans-Blot® Turbo™ Transfer System; Bio-Rad Laboratories) to nitrocellulose membranes, and subjected to Western blot analysis. Non-specific protein binding was blocked with 5% non‐fat‐dried milk (Bio‐Rad Laboratories) in Tris‐buffered saline TBS/0.05% Tween 20 (TBS‐T) for 1 h. Blots were incubated with the primary antibodies listed in Table 1. After washing, blots were incubated with appropriate secondary antibodies conjugated to horseradish peroxidase (Amersham Italia s.r.l., Milano, Italy) diluted 1:5000 in TBS-T. Bound antibodies were detected with Clarity Western ECL Substrate (Bio‐Rad Laboratories). Images were acquired with Chemidoc (Bio‐Rad Laboratories). Band densitometric analysis was performed using ImageJ software (National Institutes of Health; https://imagej.nih.gov/ij/download.html).

### Analysis of HTR-8/SVneo cell cycle after hrCNTF treatment

To examine the role of rhCNTF in cell proliferation, HTR-8/SVneo cells were treated with 50 ng/ml hrCNTF for 24 h, suspended at 1 × 10^6^ cells/ml in 1 ml PBS and centrifuged at 200 g for 5 min at RT. PBS was then removed and cells were fixed by adding 4.5 ml 70% cold ethanol (− 20 °C) overnight at 4 °C. After fixation, cells were centrifuged at 400 g for 5 min; pellets were washed 3 times with 5 ml PBS and centrifuged at 400 g for 5 min. After removing the supernatant, cells were resuspended in 1 ml propidium iodide solution (Miltenyi Biotec, Bergisch Gladbach, Germany) and incubated for 30 min at RT in the dark. Approximately 20,000 cells/sample were analysed using FacsCanto II (DB Biosciences, San Jose, CA, USA) with excitation at 488 nm and emission at 617 nm. The percentage of cells in each phase of the cell cycle was determined using FacsDiva Software (DB Biosciences).

### Wound healing assay

To investigate the role of CNTF in cell motility, HTR-8/SVneo cells grown to confluence were scratch-wounded with a sterile plastic micropipette tip. Cells were rinsed 3 times with warm medium to remove any scraped off cells in the wound and then treated/not treated with 50 ng/ml rhCNTF for 24 h. Digital images were taken at 0, 4 and 24 h. The area of the wound not occupied by cells was measured using ImageJ software. The experiment was repeated at least 3 times.

### Transwell invasion assay

Matrigel invasion assays were performed using 24-well transwell inserts with 8.0 μm pores (cat. no. 353097) coated with 100 µl of 250 µg/ml LDEV-free Matrigel (cat. no. 356234; all from Corning, NY, USA) diluted in serum-free RPMI 1640 medium according to the manufacturer’s instructions. The effects of CNTF on invasiveness were assessed by pretreating HTR-8/SVneo cells with 50 ng/ml CNTF for 24 h before seeding in the transwell chambers. In the upper chambers, 5 × 10^4^ cells were seeded on top of the Matrigel and supplemented with 200 µl serum-free RPMI 1640, with/without 50 ng/ml CNTF. The lower chambers contained 750 µl of the medium with a chemoattractant (10% FBS). After 24 h, non-invading cells and Matrigel were gently removed from the apical side of the membrane by scrubbing with a cotton swab, whereas the cells remaining on the lower surface were fixed with 100% methanol for 20 min and stained with 0.5% crystal violet for 15 min. Four non-overlapping fields of view per sample were captured at 10 × magnification and cells were counted using ImageJ software. Data were expressed as mean cell number in each of the four fields. The experiments were repeated 3 times in triplicate.

### Statistical analysis

Data are reported as mean ± standard deviation (SD). Statistical significance was analysed using Student’s *t*-test (*p* < 0.05). Statistical analyses were performed with GraphPad Prism (ver. 8) software.

## Results

### CNTF and CNTFRα were expressed in normal human placenta and in human placenta cell models (HTR-8/SVneo and BeWo)

**Fig. 1:**
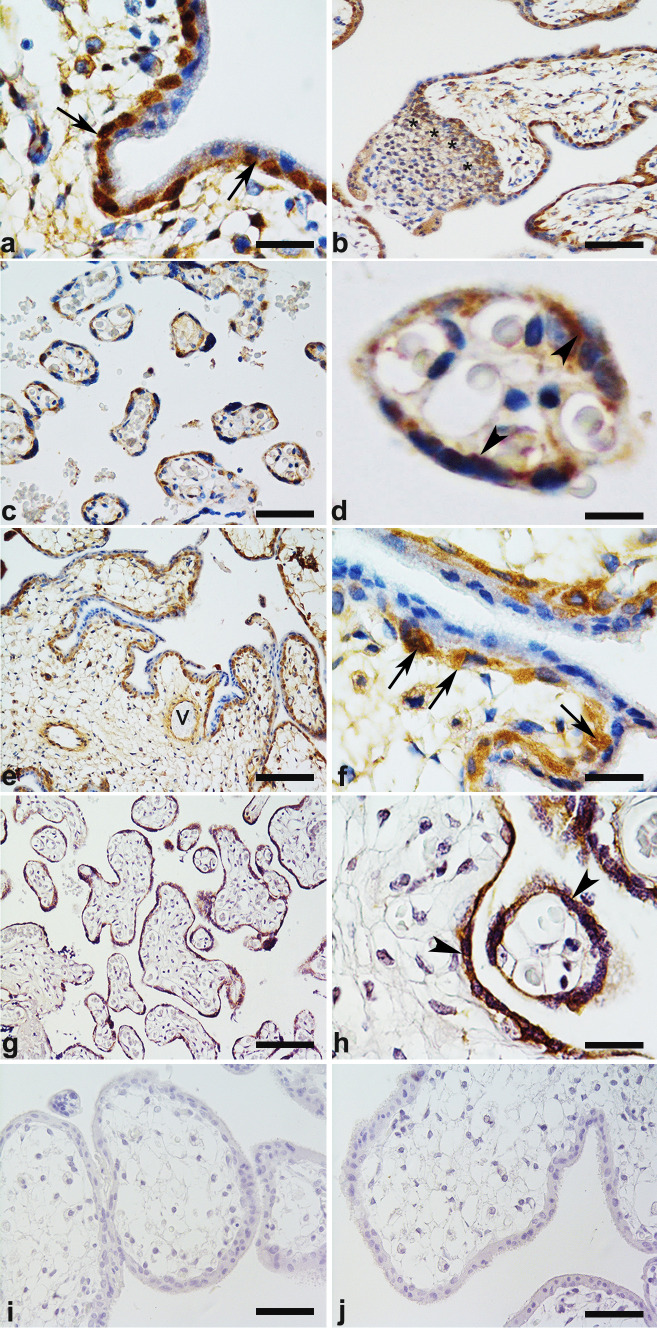
Immunohistochemistry of CNTF (**a**–**d**) and CNTFR*a* (**e**–**h**) in normal first-trimester (**a**, **b**, **e**, **f**) and third-trimester (**c**, **d**, **g**, **h**) placentas. **a** In first-trimester placental villi, CNTF is expressed in villous cytotrophoblast cells (arrows) whereas the syncytiotrophoblast is negative. **b** The extravillous cytotrophoblast cells are CNTF-positive, showing intense staining in the proximal portion of cell columns, near the villus (*). In third-trimester placental villi, only the syncytiotrophoblast (arrowheads) shows immunostaining (**c**, **d**). In (**d**), a terminal villus shows that the syncytiotrophoblast is CNTF-positive. In first-trimester placental villi, CNTFR*a* (**e**, **f**) is expressed in villous cytotrophoblast cells (arrows) whereas the syncytiotrophoblast is negative. Foetal vessels are CNTFR*a*-positive (**e**: V). **g**, **h** In third-trimester placental villi, only the syncytiotrophoblast (arrowheads) shows CNTFR*a* immunostaining. **i**, **j** Negative controls. **b** Bar = 50 mm; **c**, **e**, **g**, **i**, **j** bars = 100 mm; **a**, **f**, **h** bars = 30 mm; **d** bar = 25 mm

**Fig. 2:**
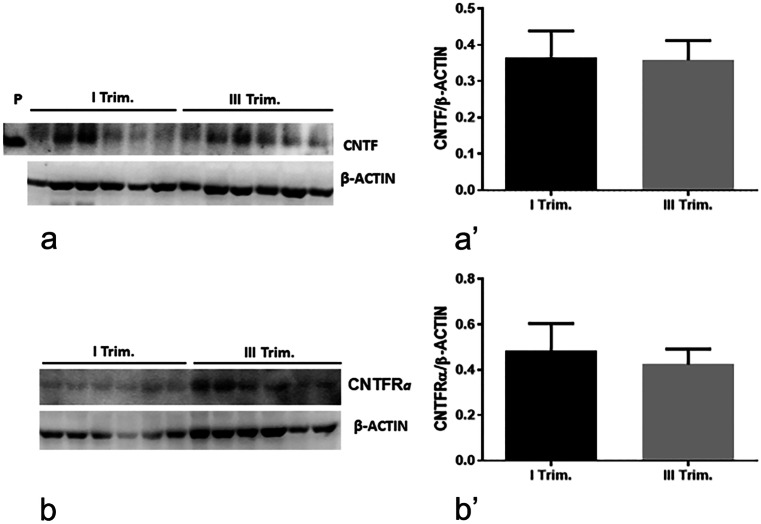
Western blots showing CNTF (**a**) and CNTFR*a* (**b**) protein expression in first- and third-trimester normal placentas. Bands (**a**, **b**) were analysed densitometrically (**a**’, **b**’). Results are expressed in arbitrary units (A.U.) and shown as histogram bars (**a**’, **b**’). CNTF and CNTFR*a* quantities were normalized using β‐actin expression. 5 ng of human hrCNTF was used as positive control (not shown). There were no significant differences in CNTF and CNTFR*a* protein expression between the first (I Trim) and third (III Trim) trimester (*n* = 12 for I Trim; *n* = 12 for III Trim)

**Fig. 3:**
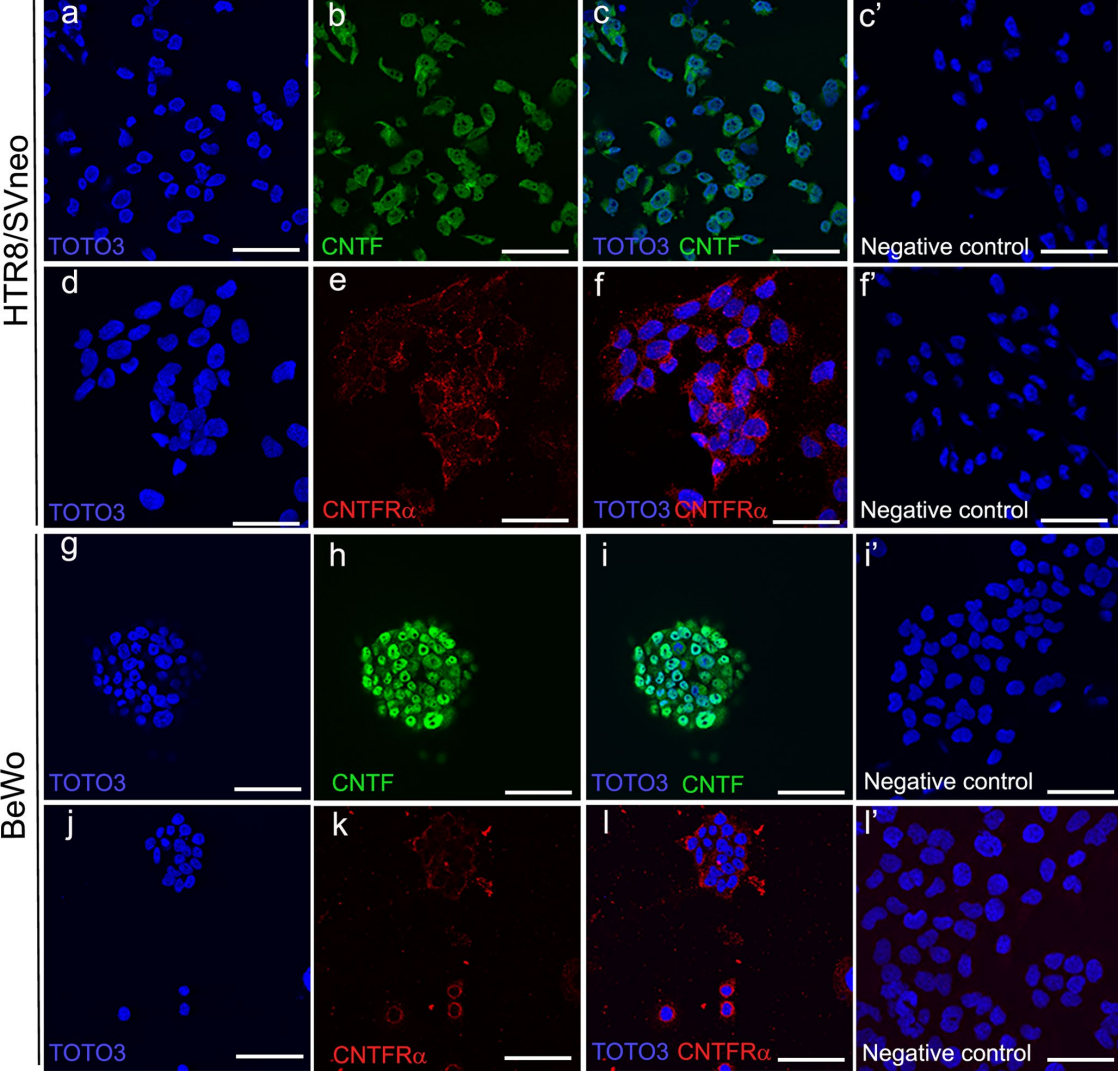
CNTF and CNTFR*a* immunofluorescence in HTR-8/SVneo (**a**–**f**) and BeWo (**g**–**l**) cells. CNTF immunostaining (green) is localized in the cytoplasm and nucleoplasm of both HTR-8/SVneo (**b**–**c**) and BeWo (**h**–**i**) cells, whereas the nucleoli are CNTF-negative. CNTFRα immunostaining (red) is localized in the cytoplasm and is especially intense in the perinuclear region of both HTR-8/SVneo (**e**–**f**) and BeWo (**k**–**l**) cells. The nuclei are stained blue. **a**–**c**, **g**–**l** Bars = 100 mm; **d**–**f** bars = 50 mm. Negative control: **c**’, **f**’, **i**’, **l**’ bars = 100 mm (HTR-8/SVneo) and 50 mm (BeWo)

In first-trimester placentas, CNTF (Fig. 1a, b) and CNTFRα (Fig. 1e, f) immunohistochemistry showed that both molecules were expressed in the cytotrophoblast layer (Fig. 1a, f: arrows), in the foetal vessels (see, for example, CNTFRα staining in Fig. 1e: V) and in the extravillous cytotrophoblast of cell columns and islands (see, for example, CNTF staining in Fig. 1b, where a column is indicated by asterisks). The syncytiotrophoblast of first-trimester placentas was negative for both CNTF and CNTFRα. In striking contrast, in third-trimester placentas, CNTF (Fig. 1c, d) and CNTFRα (Fig. 1g, h) were detected in the syncytiotrophoblast layer (Fig. 1d, h: arrowheads), whereas cytotrophoblast and endothelial cells were negative for both molecules. CNTF (Fig. 2a) and CNTFRα (Fig. 2b) were identified in the Western blots of first- and third-trimester placentas, but showed no significant differences in expression (a’, b’). By immunofluorescence analysis, HTR-8/SVneo and BeWo cells (respectively in vitro villous cytotrophoblast and syncytiotrophoblast models) were positive for both CNTF (Fig. 3 green staining) and CNTFRα (Fig. 3 red staining). In particular, CNTF was expressed in the nuclei and the cytoplasm of both cell lines (HTR-8/SVneo cells, Fig. 3a–c; BeWo cells, Fig. 3g–i), whereas CNTFRα staining was limited to the cytoplasm, especially the perinuclear region (HTR-8/SVneo cells, Fig. 3d–f; BeWo cells, Fig. 3k–l).

### Effect of CNTF on MMPs modulation

**Fig. 4:**
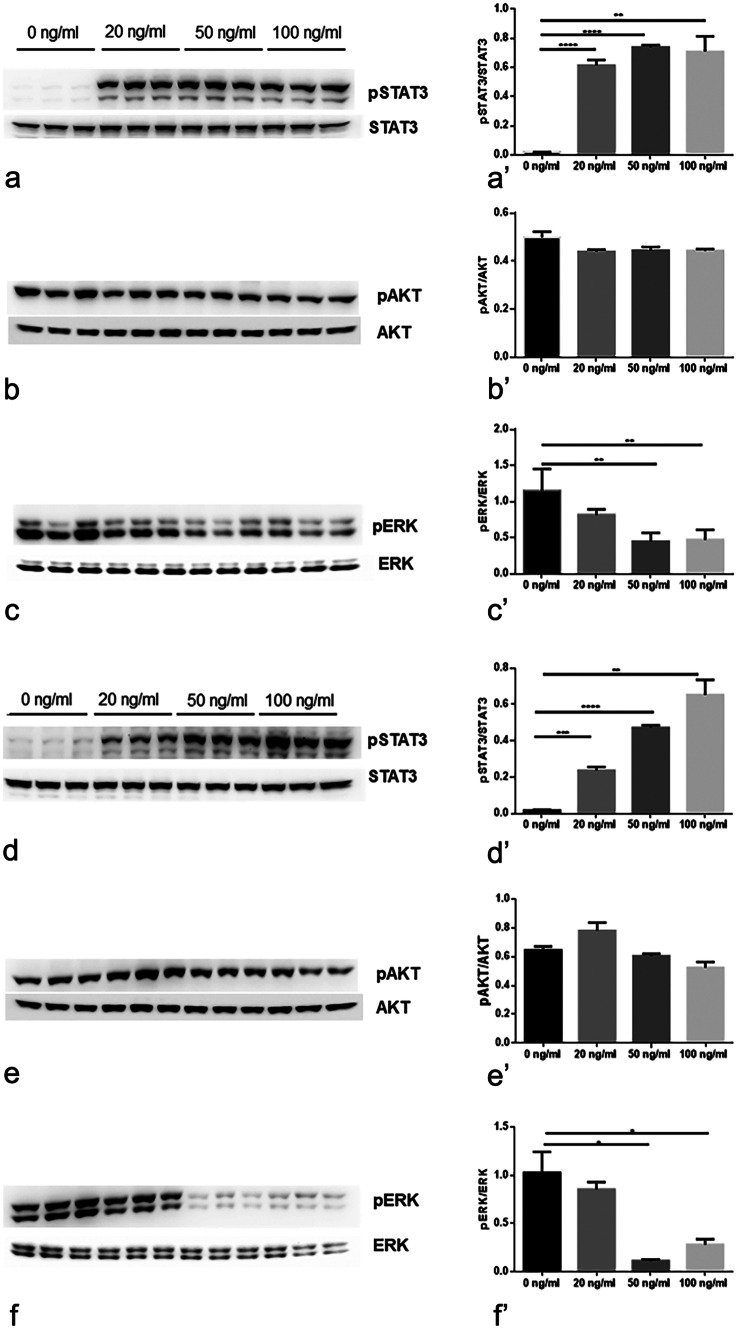
In HTR-8/SVneo and BeWo cells, CNTF induced pSTAT3 upregulation and pERK downregulation. Left, Western blots (**a**–**f**); right, histograms (**a**’–**f**’) showing band quantification. **a**, **a**’, **b**, **b**’, **c**, **c**’ HTR-8/SVneo cells and **d**, **d**’, **e**, **e**’, **f**, **f**’ BeWo cells after treatment with 20, 50 or 100 ng/ml rhCNTF. **a**’, **d**’ significant increase in pSTAT3 protein levels; **b**’, **e**’ unchanged pAKT protein levels; **c**’, **f**’ significant reduction in pERK protein levels. Results are expressed in arbitrary units (A.U.) and reported as histogram bars. pSTAT3, pAKT and pERK1/2 quantities were normalized using total STAT3, AKT and ERK1/2, respectively. Data are mean ± SD (*n* = 3). *p < 0.05, **p < 0.01, ***p < 0.001, ****p < 0.0001

**Fig. 5:**
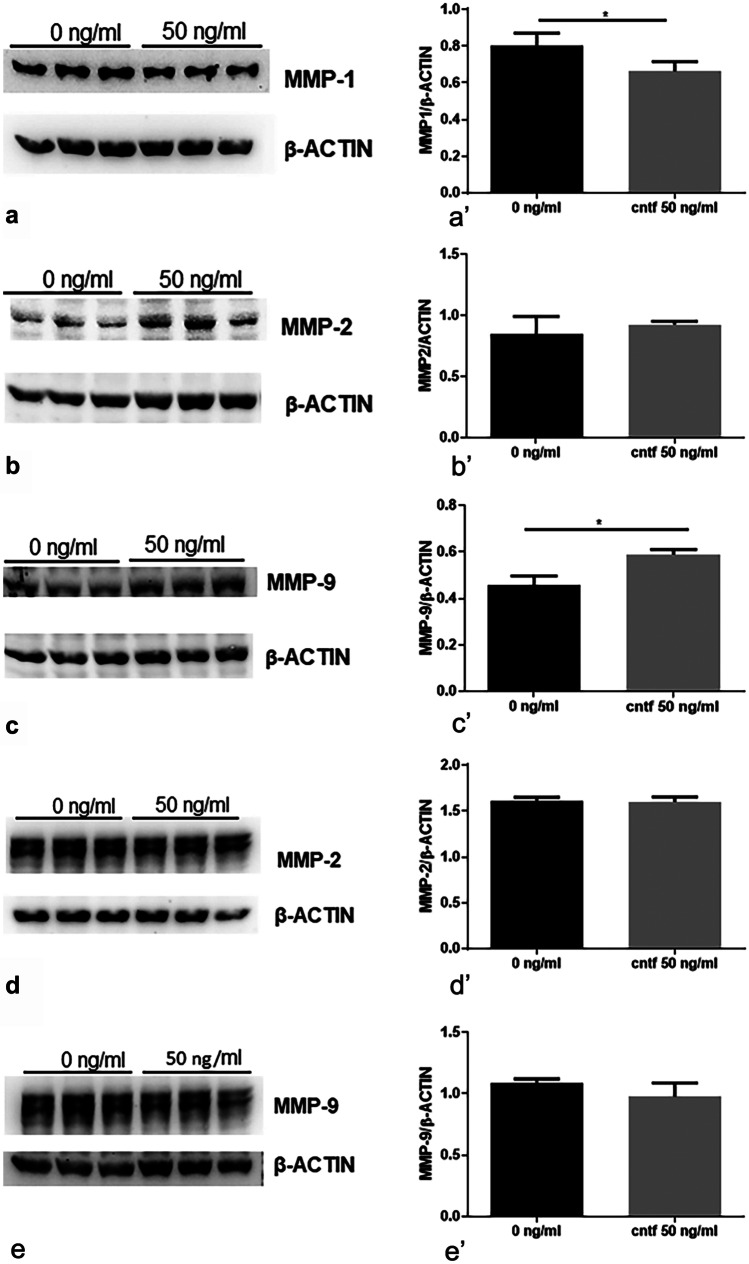
In HTR-8/SVneo cells, CNTF treatment induced MMP-1 downregulation and MMP-9 upregulation. Left, Western blots (**a**–**e**); right, histograms (**a**’–**e**’) showing band quantification. **a**, **a**’, **b**, **b**’, **c**, **c**’ HTR-8/SVneo cells and **d**, **d**’, **e**, **e**’ BeWo cells after treatment with 50 ng/ml rhCNTF. **a**’ Significant MMP-1 downregulation; **b**’ lack of significant differences in MMP-2; **c**’ significant MMP-9 upregulation; **d**’ lack of significant differences in MMP-2 protein levels; **e**’ unchanged MMP-9 protein expression. MMP-1, MMP-2 and MMP-9 quantities were normalized using β‐actin. Results are expressed in arbitrary units (A.U.) and reported as histogram bars. Data are mean ± SD (*n* = 3). *p < 0.05

**Fig. 6:**
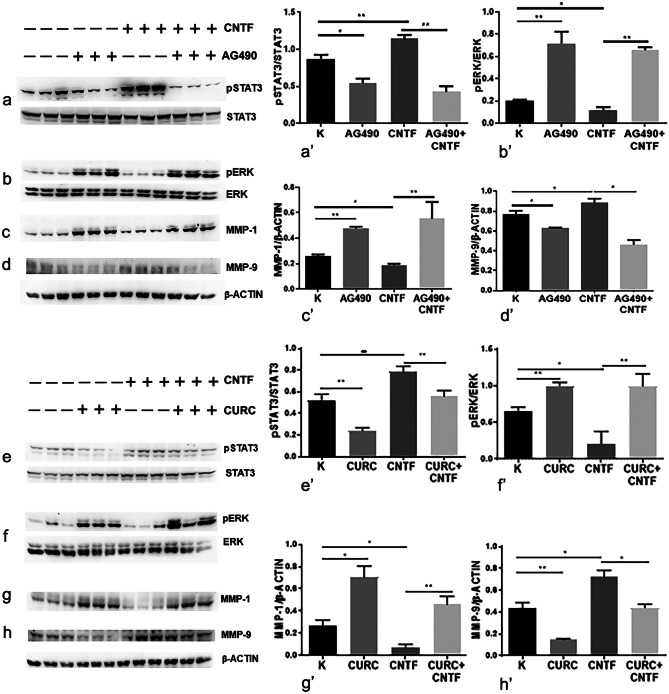
MMP-1 expression is mediated by the MAPK/ERK pathway whereas MMP-9 expression is mediated by the JAK2/STAT3 pathway. Left, Western blots (**a**–**h**); right, histograms (**a**’–**h**’) showing band quantification. Use of AG490 (**a**–**d**) and curcumin (CURC) (**e**–**h**) to inhibit the pSTAT3 pathway. (**a**’, **e**’) pSTAT3 inhibition was related to MMP-9 downregulation (**d**’, **h**’) and pERK (**b**’, **f**’) and MMP-1 (**c**’, **g**’) upregulation. pSTAT3 and pERK quantities were normalized using total STAT3 and ERK1/2, respectively. MMP-1 and MMP-9 quantities were normalized using β-actin. Results are expressed in arbitrary units (A.U.) and reported as histogram bars. Data are mean ± SD (*n* = 3). *p < 0.05, **p < 0.01

rhCNTF activated the JAK2/STAT3 pathway in a dose-dependent manner in both HTR-8/SVneo (Fig. 4a–c) and BeWo (Fig. 4d–f) cell cultures; in particular, a significantly increased pSTAT3 expression was already detected after treatment with 20 ng/ml rhCNTF (Fig. 4a’, d’). Interestingly, the CNTF treatments did not induce modulation of AKT signalling (Fig. 4b’, e’), whereas they did inhibit the MAPK/ERK pathway, with a statistically significant effect at 50 and 100 ng/ml rhCNTF (Fig. 4c’, f’). The 50 ng/ml concentration, which can activate the JAK2/STAT3 pathway and inhibit MAPK/ERK signalling, induced significant downregulation of MMP-1 (Fig. 5a, a’) and significant upregulation of MMP-9 (Fig. 5c, c’) in HTR-8/SVneo cells, but exerted no significant effect on BeWo cells (Fig. 5e, e’). Since these data suggest that MMP-1 and MMP-9 expression may be under the control of MAPK/ERK and JAK2/STAT3 signalling, respectively, we investigated the role of the two pathways in modulating MMP-1 and MMP-9 protein expression using AG490 as a JAK2 inhibitor and curcumin as a STAT3 inhibitor (Rezende et al. 2009). As expected, AG490 (Fig. 6a–d) and curcumin (Fig. 6e–h) significantly inhibited pSTAT3 expression (Fig. 6a’, e’) and increased ERK1/2 activation (Fig. 6b’, f’), resulting in MMP-1 upregulation (Fig. 6e’, g’) and MMP-9 downregulation (Fig. 6d’, h’). rhCNTF treatment induced opposite effects, suggesting that the reduced MMP-1 expression was due to suppression of MAPK/ERK signalling, whereas the increased MMP-9 expression was related to JAK2/STAT3 activation.

### CNTF did not modulate HTR-8/SVneo cell proliferation

**Fig. 7:**
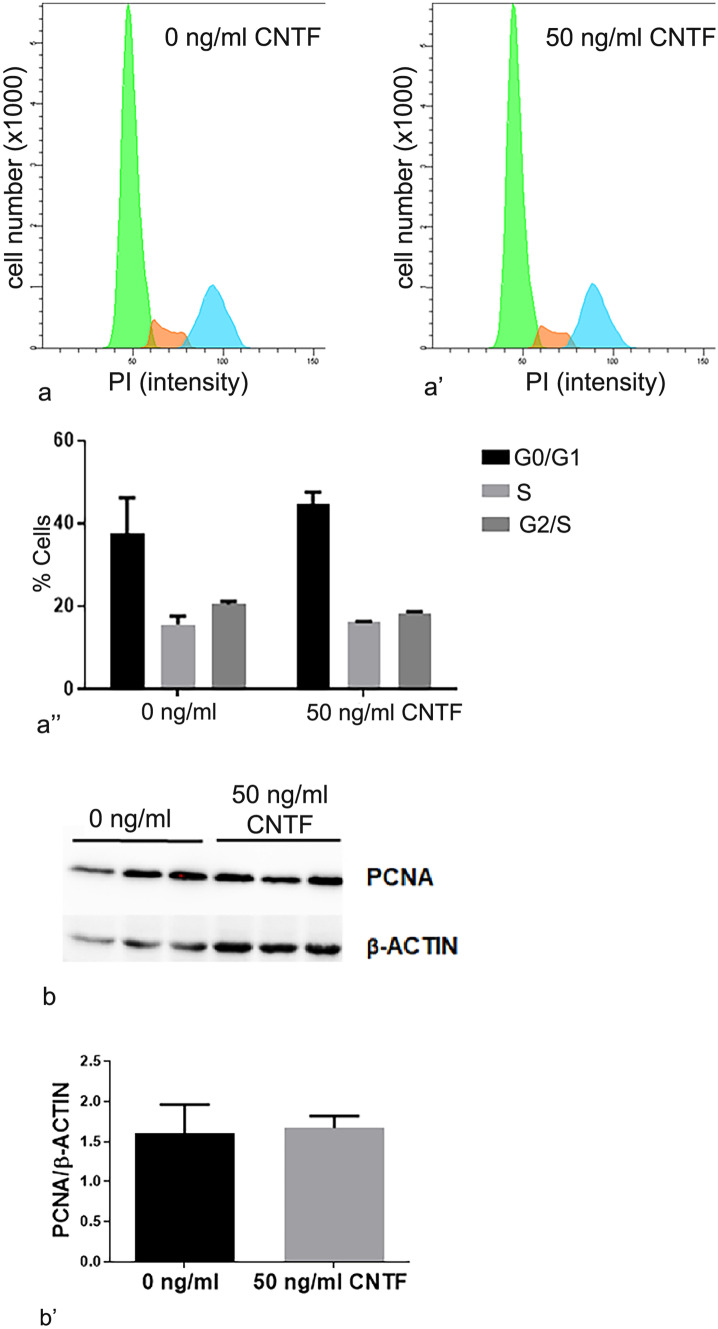
The HTR-8/SVneo cell cycle was not changed by CNTF treatment. Graphs representing untreated (**a**) and CNTF-treated (**a**’) HTR-8/SVneo cells. The area under the green curve represents the G0/G1 phases, the red curve the S phase and the blue curve the G2/M phases of the cell cycle. **a**’’ Histograms represent the percentage of the cells in each cell cycle phase and show that there were no significant differences in the cell cycle phases between treated and untreated cells. **b** PCNA Western blots of HTR-8/SVneo cells, untreated and CNTF-treated. Right, **b**’ histograms showing band quantification. There were no significant differences between untreated and CNTF-treated cells. Bands were normalized using β‐actin. Results are expressed in arbitrary units (A.U.) and reported as histogram bars. Data are mean ± SD (*n* = 3)

The cell cycle phases did not exhibit significant changes between cells treated with 50 ng/ml hrCNTF for 24 h and untreated cells (Fig. 7a). This finding was further confirmed by the analysis of PCNA expression in the Western blots, which was not significantly different in treated and untreated cells (Fig. 7b).

### CNTF did not affect the motility and invasion of HTR-8/SVneo cells

**Fig. 8:**
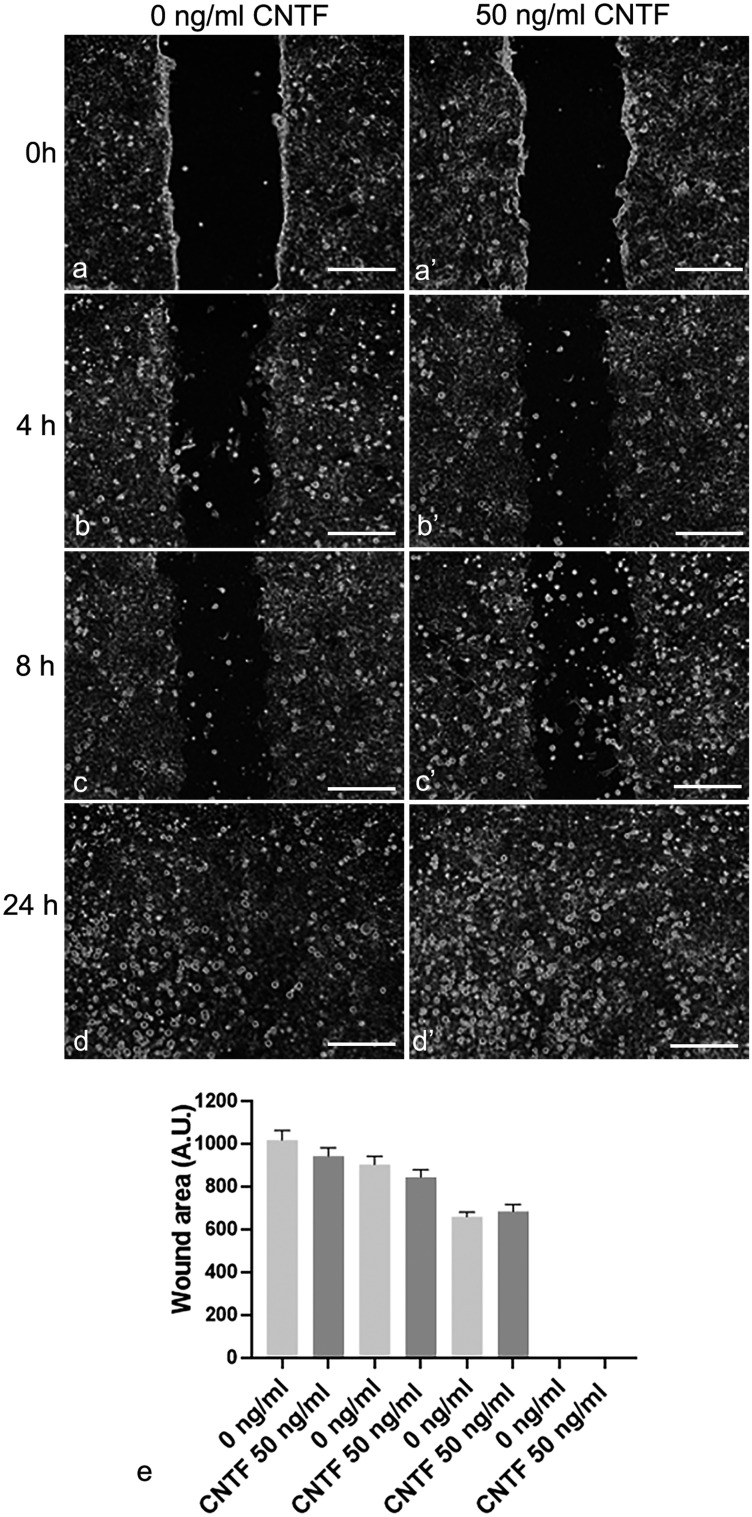
Wound closure experiments. CNTF did not affect HTR-8/SVneo cell mobility. **a**–**d**, **a**’–**d**’ Representative photographs show the effect of CNTF treatment on cell migration at different time points (0, 4, 8, 24 h). **e** The graph, representing the cell-free area, shows that CNTF treatment did not affect wound closure. In treated and untreated cells, wound closure was completed in 24 h. Results are expressed in arbitrary units (A.U.) and reported as histogram bars. Bars = 100 mm. Data are mean ± SD (*n* = 3)

**Fig. 9:**
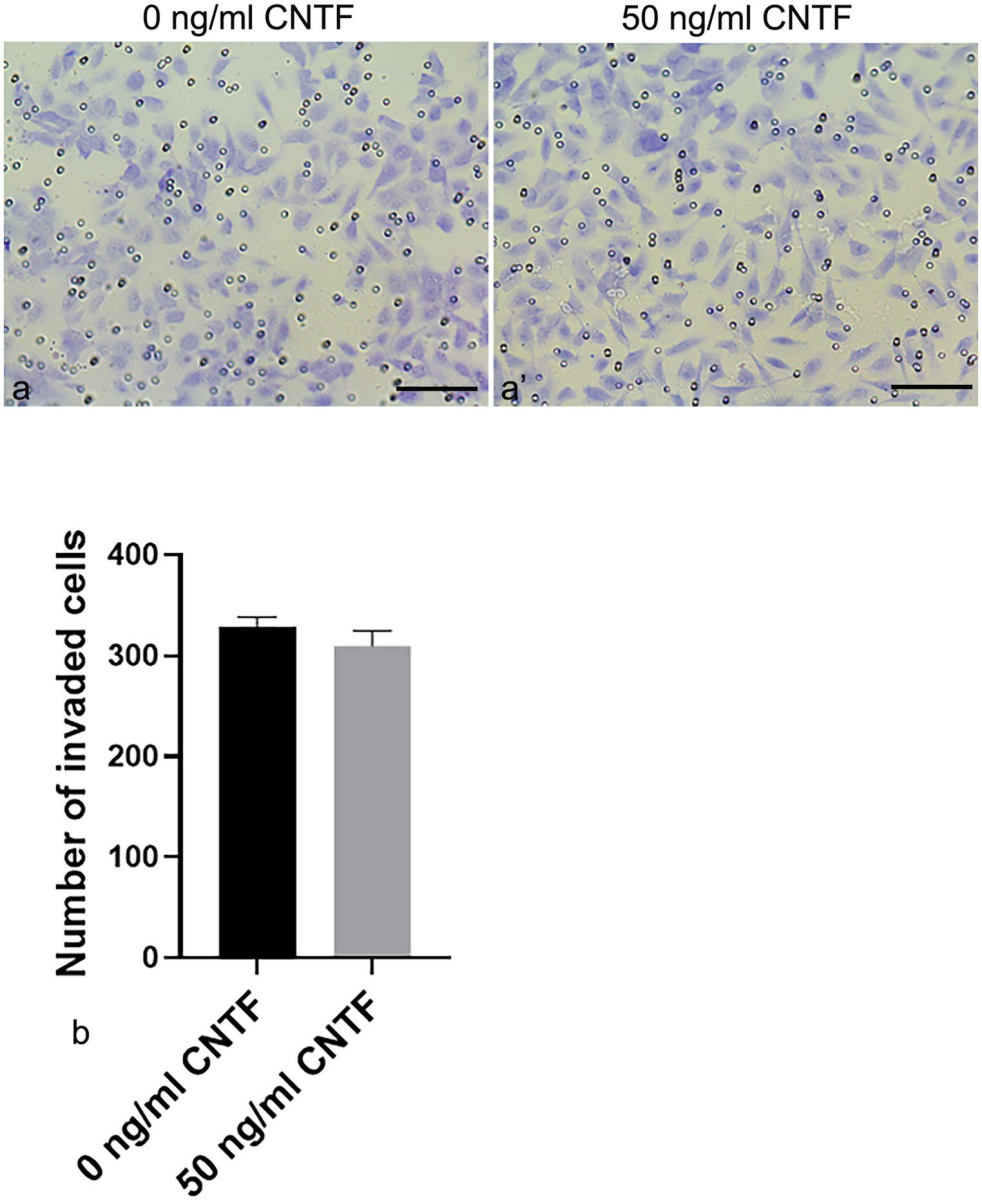
Transwell invasion assays. CNTF did not affect HTR-8/SVneo cell invasion. Representative images showing the effect of CNTF (**a**’) on HTR-8/SVneo cell invasion (**a**, **a**’). **b** The graph shows that there were no differences in invasion between treated and untreated HTR-8/SVneo cells. Invading cells are expressed as mean ± SD (*n* = 3) of invading cells/field and reported as histogram bars. Bars = 100 µm

To assess the effect of CNTF on cell migration, HTR-8/SVneo cells were treated with 50 ng/ml hrCNTF for 0, 4, 8 or 24 h (Fig. 8). The lack of significant differences in cell migration (Fig. 8e) suggested that CNTF is not involved in modulating villous cytotrophoblast migration. HTR-8/SVneo cells were also pretreated with 50 ng/ml hrCNTF for 24 h, to establish whether CNTF affects invasion properties. However, there was no significant difference in invasion ability between treated and untreated cells (Fig. 9). These data suggest that MMP-1 and MMP-9 modulation by CNTF did not alter cell motility and invasion in our in vitro model.

## Discussion

The placenta is a vital organ with key roles in foetal development. In the first stages of gestation, the cytotrophoblast differentiates into extravillous and villous. The villous cytotrophoblast gives rise to the syncytiotrophoblast (Huppertz 2008). In differentiated placental villi at the end of gestation, the trophoblast surface consists of a continuous outer syncytiotrophoblast layer beneath which lies a discontinuous layer of villous cytotrophoblast cells. The villous cytotrophoblast is the proliferating stem cell that can differentiate into the syncytiotrophoblast, forming the maternal-foetal transport barrier. In contrast, extravillous trophoblast cells lie outside the villi and form cell columns and islands, which are the result of different stages of proliferation and differentiation and whose main function is to anchor the placenta to the uterine wall (Moser et al. 2018). Thus, the villous and extravillous cytotrophoblast is a key structure for the development of the placental villous tree and play a central role in growth and invasion processes during gestation (Ferretti et al. 2007; Muhlhauser et al. 1995), whereas the syncytiotrophoblast is responsible for foetal-maternal exchanges (Huppertz 2008). In this study, we found that in the first trimester of gestation CNTF and CNTFRα are expressed in both villous and extravillous cytotrophoblast cells, whereas in the third trimester their expression is confined to the syncytiotrophoblast. We propose that such consistent expression in structures vital for placental growth makes CNTF a constitutive protein with a potential role in the maintenance of placental morphology. A paracrine role for this cytokine linked to placental tissue remodelling during gestation cannot be excluded. In particular, the presence of CNTFRα in the foetal vessels suggests a possible role of CNTF in vascular remodelling as demonstrated in other tissues including the retina, where CNTF has been shown to exert an anti-angiogenic effect on endothelial cell sprouting through activation of the JAK/STAT3/SOCS3 signalling (Bucher et al. 2016).

In addition, it is also conceivable that in some conditions, CNTF may be secreted into the maternal circulation, thus acting as a hormone, as described previously (Akahori et al. 2010; Bienertova-Vasku et al. 2013).

It has clearly been documented that CNTF can modulate MMPs in human tissues (Chen et al. 2016) and that the invasive capacity of the trophoblast is strongly associated with its ability to produce and secrete MMPs (Fisher et al. 1989; Librach et al. 1991; Vegh et al. 1999). Here, we suggest that the presence of CNTFRα in the villous and extravillous cytotrophoblast may be related to the modulation of MMP-1 and MMP-9 through the action of CNTF. Interestingly, CNTF appears to regulate their expression through two different pathways. Our findings provide the first evidence of endogenous CNTF production, whose function seem to be the maintenance of placental tissue homeostasis. Notably, we demonstrated that MMP-1 downregulation was mediated by inhibition of MAPK/ERK signalling, whereas MMP-9 upregulation was mediated by activation of the JAK2/STAT3 pathway, in line with a recent report showing MMP-9 modulation by JAK2/STAT3 signalling in HTR-8/SVneo cells (Zong et al. 2016). We also demonstrated that CNTF did not affect cell proliferation, mobility and invasion, but only the balance of MMPs secretion.

So, MMPs effect was not detected in mobility and invasion processes probably due to the compensatory action of MMP-9 and MMP-1.

Collagen is a major substrate for MMPs and type I, III, IV and V collagens are expressed in human placenta (Oefner et al. 2015; Sati et al. 2008). In particular, type IV is chiefly expressed in the basal membrane of the placental villus, under the villous cytotrophoblast and around foetal vessels, whereas types I and III are localized in the interstitial stroma of foetal villi and in the extravillous trophoblast (Oefner et al. 2015; Sati et al. 2008). MMP-1 is an interstitial collagenase that degrades various interstitial collagens, including types I and III, thus playing a role in extravillous trophoblast invasion of the uterine wall. In contrast, MMP-9 is a type IV collagenase that principally degrades collagen type IV, in this respect it being similar to MMP-2 (Klein and Bischoff 2011). Notably, complete inhibition of in vitro trophoblast invasion by an MMP-9 antibody has suggested a key role for it in cell invasion processes (Librach et al. 1991). The proliferative ability of the villous cytotrophoblast and the extravillous cytotrophoblast are ensured by closeness to the basal membrane, where they form a compact cluster of cells. When villous cytotrophoblast cells leave the basal membrane, they lose their polarity and acquire an invasive phenotype that allows interaction with the surrounding extracellular matrix molecules, such as MMPs (Benirschke et al. 2006). Altogether, these data suggest that CNTF is involved in controlling the first phases of villous and extravillous cytotrophoblast migration by promoting collagen IV degradation by MMP-9 and by inhibiting cell mobility via MMP-1 downregulation. It is conceivable that MMP-9 upregulation by CNTF induces collagen type IV degradation which would be followed by a breach in the basal membrane and the switch of trophoblast cells to the invasive phenotype. The hypothesis is consistent with studies demonstrating that MMP-9 downregulation in the trophoblast plays an important role in PE onset, since a shallow extravillous trophoblast invasion hampers the transformation of uterine spiral arteries in the placental bed (Chen and Khalil 2017). In conclusion, we demonstrate that CNTF should be considered a constitutive placental cytokine acting in structures which are essential for human placental development and function, and that it plays a central role in modulating MMP-1 and MMP-9 expression by two different signalling pathways.
