# Correction to: Modulation of matrix metalloproteases by ciliary neurotrophic factor in human placental development

**DOI:** 10.1007/s00441-023-03771-9

**Published:** 2023-04-22

**Authors:** Giovanni Tossetta, Sonia Fantone, Elena Marinelli Busilacchi, Nicoletta Di Simone, Stefano R. Giannubilo, Giovanni Scambia, Antonio Giordano, Daniela Marzioni

**Affiliations:** 1 Department of Experimental and Clinical Medicine, Università Politecnica Delle Marche, 60126 Ancona, Italy; 2 Clinica Di Ostetricia E Ginecologia, Azienda Ospedaliero Universitaria Ospedali Riuniti Di Ancona, 60123 Ancona, Italy; 3 Department of Clinical and Molecular Sciences, Università Politecnica Delle Marche, Ancona, Italy; 4 Hematology Unit, AUO Ospedali Riuniti Di Ancona, 60123 Ancona, Italy; 5 Department of Biomedical Science, Humanitas University, Via Rita Levi Montalcini 4, 20090 Pieve Emanuele, Milan, Italy; 6 Humanitas Clinical and Research Center-IRCCS, Via Manzoni56, 20089 Rozzano, Italy; 7 Department of Clinical Sciences, Università Politecnica Delle Marche, Salesi Hospital, 60123 Ancona, Italy; 8 U.O.C. Di Ostetricia E Patologia Ostetrica, Dipartimento Di Scienze Della Salute Della Donna, Fondazione Policlinico Universitario A. Gemelli IRCCS, del Bambino E Di Sanità Pubblica, 00168 Rome, Italy; 9 Istituto Di Clinica Ostetrica E Ginecologica, Università Cattolica del Sacro Cuore, 00168 Rome, Italy

**Correction to: Cell and Tissue Research (2022) 390:113–129** https://doi.org/10.1007/s00441-022-03658-1

The authors regret that Fig. 2 showed in the article was the wrong one.

The original article has been corrected.

